# Unravelling the mechanisms of Type 1A topoisomerases using single-molecule approaches

**DOI:** 10.1093/nar/gkab239

**Published:** 2021-05-08

**Authors:** Dian Spakman, Julia A M Bakx, Andreas S Biebricher, Erwin J G Peterman, Gijs J L Wuite, Graeme A King

**Affiliations:** Department of Physics and Astronomy, and LaserLaB Amsterdam, Vrije Universiteit Amsterdam, De Boelelaan 1081, 1081 HV, Amsterdam, The Netherlands; Department of Physics and Astronomy, and LaserLaB Amsterdam, Vrije Universiteit Amsterdam, De Boelelaan 1081, 1081 HV, Amsterdam, The Netherlands; Department of Physics and Astronomy, and LaserLaB Amsterdam, Vrije Universiteit Amsterdam, De Boelelaan 1081, 1081 HV, Amsterdam, The Netherlands; Department of Physics and Astronomy, and LaserLaB Amsterdam, Vrije Universiteit Amsterdam, De Boelelaan 1081, 1081 HV, Amsterdam, The Netherlands; Department of Physics and Astronomy, and LaserLaB Amsterdam, Vrije Universiteit Amsterdam, De Boelelaan 1081, 1081 HV, Amsterdam, The Netherlands; Institute of Structural and Molecular Biology, University College London, Gower Street, London WC1E 6BT, UK

## Abstract

Topoisomerases are essential enzymes that regulate DNA topology. Type 1A family topoisomerases are found in nearly all living organisms and are unique in that they require single-stranded (ss)DNA for activity. These enzymes are vital for maintaining supercoiling homeostasis and resolving DNA entanglements generated during DNA replication and repair. While the catalytic cycle of Type 1A topoisomerases has been long-known to involve an enzyme-bridged ssDNA gate that allows strand passage, a deeper mechanistic understanding of these enzymes has only recently begun to emerge. This knowledge has been greatly enhanced through the combination of biochemical studies and increasingly sophisticated single-molecule assays based on magnetic tweezers, optical tweezers, atomic force microscopy and Förster resonance energy transfer. In this review, we discuss how single-molecule assays have advanced our understanding of the gate opening dynamics and strand-passage mechanisms of Type 1A topoisomerases, as well as the interplay of Type 1A topoisomerases with partner proteins, such as RecQ-family helicases. We also highlight how these assays have shed new light on the likely functional roles of Type 1A topoisomerases *in vivo* and discuss recent developments in single-molecule technologies that could be applied to further enhance our understanding of these essential enzymes.

## INTRODUCTION

**Figure 1.**
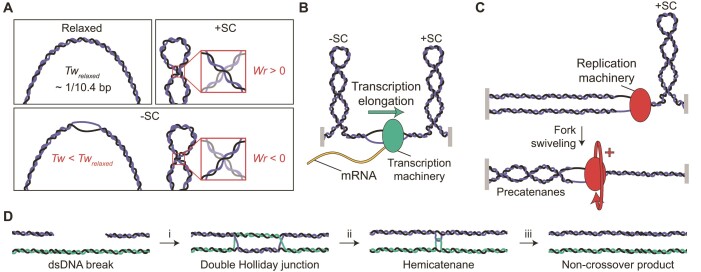
Overview of DNA topology and its interplay with genomic processes. (**A**) The influence of torsional stress on the structure of DNA. Upper left: In torsionally relaxed DNA, the linking number is equal to the twist (*Tw*), which is ∼1 turn/10.4 bp (referred to here as *Tw*_relaxed_). Upper right: Structure of positively supercoiled (+SC) DNA. At low tensions (typically below ∼3 pN), the torsional stress is stored only as writhe (*Wr* >0), through the formation of left-handed plectonemes. Lower: Structure of negatively supercoiled (-SC) DNA. The torsional stress can be stored as both twist (*Tw* < *Tw*_relaxed_) or writhe (*Wr* <0), even at low tensions (<1 pN). Changes in twist can lead to the formation of denatured, underwound structures (which often exhibit a left-handed form, e.g. L-DNA), whereas negative writhe yields right-handed plectonemes. (**B**) The Twin-Domain model for transcription. Due to the fact that genomic DNA is (locally) torsionally constrained (depicted here by grey blocks), translocation of the transcription machinery results in discrete domains of negatively supercoiled and positively supercoiled DNA behind and ahead of the transcription bubble, respectively. (**C**) Formation of precatenanes during replication. Progressive strand separation by the replication machinery leads to the accumulation of positive supercoils ahead of the replication fork, which can be relaxed by rotation of the replication fork (fork swiveling). This results in entwinement of the two daughter strands (in the form of precatenanes). (**D**) Generation and resolution of DNA entanglements during DNA repair. A dsDNA break can be repaired without crossover in a three-step process: (i) Homologous recombination results in the formation of a double Holliday junction; (ii) The double Holliday junction is converted into a hemicatenane via the concerted action of a TopoIII topoisomerase and a RecQ-family helicase (known together as the dissolvasome); and (iii) The hemicatenane is resolved by the dissolvasome into two separate dsDNA molecules.

Many genomic processes, including DNA replication, transcription and repair, result in torsional stress being exerted on DNA. Since double-stranded (ds)DNA is often torsionally constrained *in vivo*, either through a circular genome or the presence of local topological barriers (e.g. nucleosomes), such torsional stress can lead to local changes in DNA topology. The topology of DNA is described by the linking number (*Lk*), which is the sum of the twist (the number of helical turns in the DNA) and the writhe (the number of times the double helix crosses itself) (1,2). Torsionally relaxed DNA has no writhe and a twist of ∼10.4 bp/turn. However, torsional stress can induce DNA supercoiling, which corresponds to either the formation of writhe or a change in the twist relative to relaxed DNA (Figure 1A). Supercoiling can be positive or negative, depending on whether the torsional stress induces overwinding or underwinding, respectively. Under physiological conditions, positively supercoiled DNA typically adopts a plectonemic structure, in which the torsional stress is stored as writhe (3–8). While changes in twist can additionally occur in positively supercoiled DNA (through the formation of P-DNA), this generally only arises at elevated tensions (e.g. >3 pN) (3,9–12). In contrast, changes in both twist and writhe often occur in negatively supercoiled DNA, even in the absence of force, depending on the DNA sequence and the extent of supercoiling (3–8,10–18).

*In vivo*, both positive and negative supercoiling are frequently generated. For example, the transcription machinery induces positively supercoiled DNA ahead and negatively supercoiled DNA behind the transcription bubble, a phenomenon known as the Twin-Domain model (Figure 1B) (19). On average, however, the genome of most organisms is slightly negatively supercoiled. This is thought to aid genomic processes as it facilitates DNA bending and increases the probability of strand separation (20). In other genomic processes, torsional stress can lead to the entanglement of two DNA molecules. For example, translocation of the replication machinery requires the replication fork to swivel to counteract the accumulation of positive supercoiling ahead of the fork. Consequently, the two daughter DNA strands can become entangled, resulting in the formation of (pre)catenane structures (Figure 1C) (21). Entanglements can also arise during DNA repair through homologous recombination, where strand invasion leads to a branched DNA structure known as a double Holliday junction. This structure can then be converted to a hemicatenane (in which two dsDNA molecules are linked via a single-stranded crossover) through enzyme-mediated branch migration (Figure 1D) (22–28).

**Figure 2.**
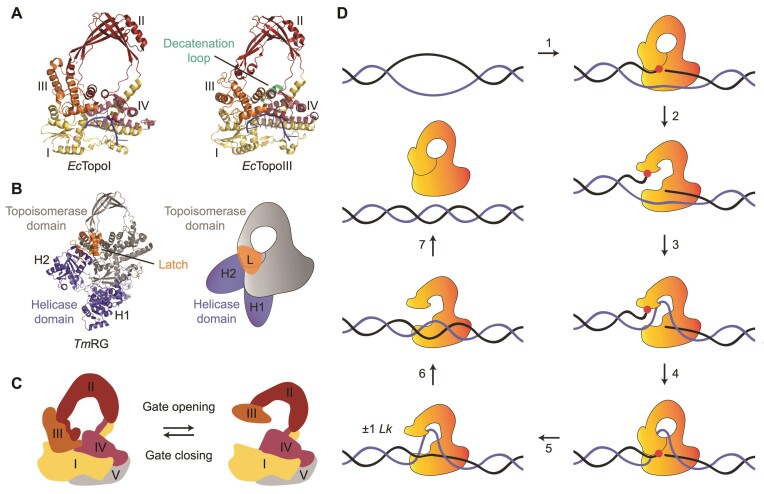
Structure and catalytic cycle of Type 1A topoisomerases. (**A**) Crystal structures of *Ec*TopoI (left, PBD 1MW8) (93) and *Ec*TopoIII (right, PDB 1I7D) (92) in a closed conformation. The four domains of the toroidal fold (domains I-IV) are indicated. Both crystal structures show an ssDNA molecule (blue) bound to the ssDNA binding site of domain I, III and IV. The decatenation loop, which is present in *Ec*TopoIII, but not in *Ec*TopoI, is highlighted in green. (**B**) Crystal structure (left, PDB 4DDU) (80) and schematic (right) of *Tm*RG, with the topoisomerase and helicase domains shown in grey and purple, respectively. The helicase domain consists of two subunits, H1 and H2, with the latter containing a latch domain (highlighted in orange). (**C**) Schematics of the closed and open conformational states of Type 1A topoisomerases, showing the four domains of the toroidal fold, along with the CTD (domain V). The schematic is based on the crystal structure of full-length *Ec*TopoI (PDB 3PWT). (**D**) Proposed model of key steps of the catalytic cycle of Type 1A topoisomerases during relaxation of supercoiled DNA. 1. The enzyme binds, in a closed conformation, to a local region of ssDNA (G-segment, black) and cleaves the backbone (via a transesterification reaction) to form an enzyme-bridged ssDNA gate. The transesterification site is highlighted by the red dot. 2. The enzyme undergoes a conformational change, resulting in gate opening. 3. A second DNA strand (T-segment, purple) enters the central cavity of the enzyme via the gate. 4. The enzyme-bridged ssDNA gate closes. 5. The ssDNA backbone is re-ligated and the enzyme adopts an open conformation. This results in a change of ±1 *Lk*. 6. The T-segment is released from the cavity. 7. The enzyme can either unbind from the DNA (in a closed conformation) or undergo further catalytic cycles. Note that this catalytic cycle is also relevant for decatenation, except that the T-segment, in that case, would come from a different DNA molecule and is therefore likely further away from the G-segment.

The topological state of DNA is regulated *in vivo* by enzymes known as topoisomerases. These enzymes are essential for both maintaining supercoiling homeostasis and disentangling (i.e. decatenating) entwined DNA strands (29,30). The first topoisomerase to be identified (initially termed ω protein) was discovered in *Escherichia coli* by James Wang in 1971 and is now known as Topoisomerase I (*Ec*TopoI, Figure 2A) (31). Since then, many different topoisomerases have been found in all three domains of life (32). Topoisomerases change the topology of DNA by transiently cleaving either one or two strands of the DNA phosphate backbone through a transesterification reaction, and can be classified into two families, Type 1 and Type 2 (32). Type 2 topoisomerases cleave both strands of the double helix and change the topology of DNA in steps of ±2 *Lk* in an ATP-dependent process (33–35). In contrast, Type 1 topoisomerases are ATP-independent (with the exception of reverse gyrases, see later) and make a transient break in one of the DNA backbones (36,37). Type 1 topoisomerases can be subdivided according to their structure and reaction mechanisms into three subfamilies: Type 1A, Type 1B and Type 1C (32,38). The first two subfamilies exist in all three domains of life (32), whereas the latter has thus far only been found in the Archaeon species *Methanopyrus kandleri* (39,40). Type 1B and Type 1C enzymes regulate DNA topology via a swivel mechanism that facilitates relaxation of DNA supercoiling in steps of ±*n Lk* (40–42). In contrast, Type 1A topoisomerases act via an enzyme-bridged strand-passage mechanism that can both change the supercoiling density of DNA and (de)catenate DNA in steps of ±1 *Lk* (36,37,43).

Since Type 1A topoisomerases are found in nearly all living organisms (32) and many exhibit RNA topoisomerase activity (44–47), it has been suggested that this family may have played an important role in early evolution. Furthermore, these enzymes are distinct from those in other topoisomerase families in that they require single-stranded (ss)DNA (or RNA) for their catalytic activity (48,49). Over the past 40 years, a wealth of biochemical and structural studies have shed significant insight into the molecular interactions and cellular functions of Type 1A topoisomerases, as discussed in several excellent reviews (38,50–55). In the current article, however, we explore how our mechanistic understanding of these vital enzymes has advanced greatly in recent years through the application of single-molecule assays based on magnetic tweezers, optical tweezers, atomic force microscopy (AFM) and single-molecule Förster resonance energy transfer (smFRET). These techniques allow detection of transient intermediate states and real-time dynamic processes that are often obscured by ensemble averaging in bulk biochemistry assays. We discuss how single-molecule assays have advanced our understanding of the catalytic mechanism of Type 1A topoisomerases, as well as the interaction of these enzymes with partner proteins. Additionally, we explore the future potential of single-molecule approaches to shed new light on the wider functional role of Type 1A topoisomerases. To facilitate these discussions, we first outline the key structural features of these enzymes and provide an overview of the most commonly used single-molecule techniques to study Type 1A topoisomerases.

## CLASSIFICATION AND FUNCTION OF TYPE 1A TOPOISOMERASES

Type 1A topoisomerases are typically further classified into three subgroups (32). The two main subgroups are TopoI and TopoIII, named after the *E. coli* prototypes *Ec*TopoI and *Ec*TopoIII, respectively (Figure 2A). While both TopoI and TopoIII enzymes can relax supercoiled DNA and (de)catenate DNA, TopoI enzymes are more efficient at relaxing supercoils (49,56) and play a key role in removing excess negative supercoiling during transcription (57,58). In contrast, TopoIII enzymes are more effective at (de)catenating DNA (49,59), and their activity is vital for removing entangled DNA structures created as a result of replication and recombination (28,60–64). TopoI enzymes are found in all bacteria, but not in archaea or eukaryotes, whereas TopoIII enzymes are present in some bacteria, most archaea and all eukaryotes (32). In eukaryotes, TopoIII enzymes are active in a tight complex with one or more evolutionary conserved OB-fold regulatory proteins (65–68). *Saccharomyces cerevisiae* (*Sc*)TopoIII, for example, forms a heterodimer with a RecQ-mediated genome instability (RMI) protein termed Rmi1 (65). Higher eukaryotes typically encode two TopoIII enzymes, known as TopoIIIα and TopoIIIβ (32). TopoIIIα is always found in a complex (known as TRR) with two RMI proteins (RMI1 and RMI2) (66,67,69), while TopoIIIβ exists in complex with another OB-fold protein named TDRD3 (68). These OB-fold proteins play an important role in modulating the activity of the topoisomerase. For example, Rmi1 and RMI1 promote the decatenation activity of *Sc*TopoIII and TopoIIIα, respectively (25,26,67,70), while TDRD3 is thought to recruit TopoIIIβ to actively transcribed genomic regions (71) and subsequently stimulate topoisomerase activity (45).

Many TopoIII enzymes (including *Ec*TopoIII, *Sc*TopoIII-Rmi1 and TRR) are able to form a complex with RecQ-family helicases (RecQ, Sgs1 and BLM in *E. coli*, *S. cerevisiae* and higher eukaryotes, respectively) (72). This complex, known as the ‘dissolvasome’, catalyzes the dissolution of late-replication and recombination intermediates, such as (pre)catenanes and double Holliday junctions (Figure 1C and D) (22–28,62,63,72,73). The latter is resolved without chromosomal crossover in a two-step process that involves convergent branch migration (resulting in a hemicatenane), followed by decatenation of the two conjoined DNA molecules (Figure 1D). In a topologically closed system, both steps require the concerted action of a RecQ helicase and a TopoIII topoisomerase. In the first step, the ATP-dependent unwinding activity of the helicase drives branch migration, while the topoisomerase enhances the activity of the helicase (24) and releases the build-up of torsional stress (23,26,27). In the second step, the ATP-dependent activity of the helicase is thought to provide sufficient ssDNA for the topoisomerase to bind to and decatenate the entangled strands (24–26). The dissolvasome often acts in concert with additional ssDNA binding proteins, such as SSB in prokaryotes and RPA in eukaryotes (28). Furthermore, the human TRR complex has been reported to cooperate with the ATP-dependent dsDNA translocase PICH (74,75), leading to the formation of positively supercoiled DNA (75).

The coordinated action of human PICH and TRR to generate positively supercoiled DNA is reminiscent of the third subgroup of Type 1A topoisomerases, namely reverse gyrases (RGs). RGs can generate positively supercoiled DNA in an ATP-dependent process, as well as being able to relax negative supercoils (76). This unusual activity is possible because these enzymes contain both a Type 1A topoisomerase domain and a helicase domain, the latter of which is structurally similar to superfamily 2 helicases (Figure 2B) (77–80). RGs are found in all hyperthermophilic and some thermophilic organisms, but not in mesophiles (81,82), suggesting a specific role of RGs at high temperatures (83,84). Some (hyper)thermophilic organisms, such as *Sulfolobus solfataricus*, encode two types of RGs (85–87), referred to here as RG1 and RG2. In the case of *S. solfataricus* (*Sso*), these enzymes can be distinguished by the strength of the coupling between their topoisomerase and helicase domains (85–88). This coupling is weak in *Sso*RG1, and as a result, this enzyme can relax negatively supercoiled DNA in the absence of ATP. In contrast, the two domains are strongly coupled in *Sso*RG2, and thus ATP is required for both supercoil relaxation and the introduction of positive supercoiling.

## STRUCTURE AND GATE OPENING OF TYPE 1A TOPOISOMERASES

Despite their difference in biological activity, the subfamilies of Type 1A topoisomerases share a highly conserved core structure consisting of four domains (termed I-IV, where domain I is also known as the Topoisomerase-Primase (TOPRIM) domain (89)). Together, these four domains form a toroidal structure (67,90–94), which can change its conformation between an open and closed state (Figure 2A and C) (95–99). This conformational change provides the foundation of the catalytic cycle of these enzymes, which involves the following key steps (Figure 2D). First, the topoisomerase binds to a segment of ssDNA (called the gated (G-)segment) via domains I, III and IV, and cleaves the ssDNA via a transesterification reaction between the 5’-end of the DNA and an active tyrosine in domain III (92,93,100–102). The 3’-end of the cleaved ssDNA is simultaneously held via electrostatic interactions with domain IV. Next, domain III moves away from domains I and IV, resulting in the opening of the enzyme-bridged gate in the ssDNA. This allows a second strand of DNA (called the transfer (T-)segment) to access the central cavity of the toroidal fold (91). The central cavity has been reported to have a diameter of approximately 27.5 Å for *Ec*TopoI (90), 25 Å for *Thermatoga maritima* (*Tm*)TopoI (94) and 26 Å for human TopoIIIα (67), which suggests that it can accommodate either ssDNA or dsDNA (67,90,91,94,96). Following this, the enzyme-bridged ssDNA gate closes, and the backbone is re-ligated (92). Finally, the enzyme adopts an open-gate conformation, allowing the T-segment DNA to be released from the cavity (93). The completed cycle results in a change of ±1 *Lk* (43), after which the enzyme can either repeat this cycle or unbind from the DNA (Figure 2D).

The catalytic activity of Type 1A topoisomerases is dependent on the presence of divalent metal ions. Binding of magnesium ion(s) to a DxD motif within the TOPRIM domain (103) is essential for the conformational changes required for Type 1A topoisomerase activity (98,103–105) and re-ligation of the G-segment after strand passage (99,106–108). Furthermore, magnesium is thought to play a role in (non-covalent) ssDNA binding (98,99) and subsequent ssDNA cleavage (98,104,109). Alongside the toroid core, Type 1A topoisomerases also contain a carboxyl (C)-terminal domain (CTD) (domain V, Figure 2C) (110), which varies in size and sequence between species (51). The CTD of many Type 1A topoisomerases contains putative zinc-binding cysteine motifs, which have been proposed to help facilitate strand passage during supercoil relaxation by interacting with either the G- or T-segment (110–113).

## SINGLE-MOLECULE APPROACHES TO STUDY TYPE 1A TOPOISOMERASES

**Figure 3.**
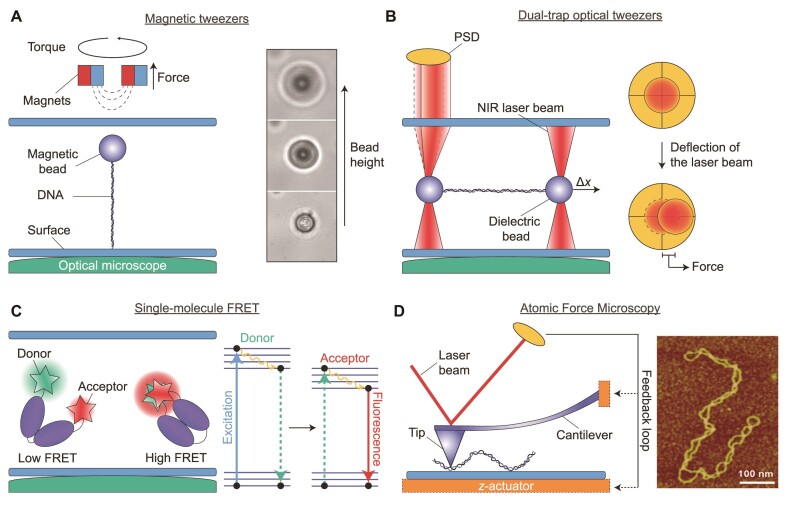
Schematic overview of single-molecule techniques used to study Type 1A topoisomerases. (**A**) A typical magnetic tweezers assay. Left: A DNA molecule is tethered between a glass surface and a paramagnetic bead. A magnet placed above the sample chamber allows force to be applied to the bead (and thus the DNA). When using two horizontally-aligned magnets (as shown here), torsional stress can also be generated in the DNA by rotating the magnets (and therefore the bead). Right: Sample bright-field images showing the change in diffraction pattern of the bead as a function of magnet height, from which the DNA length can be determined. Right panel adapted from Figure 2D of (131). (**B**) A typical dual-trap optical tweezers assay. Left: A DNA molecule is tethered between two dielectric beads trapped by strongly focused near-infrared (NIR) laser beams. The DNA molecule can be extended by displacing one of the beads (Δ*x*), resulting in an applied force. The force leads to a small deflection of the laser beam, which can then be measured by back-focal plane imaging on a position-sensitive detector (PSD) (yellow). Right: Top-down schematic view of the PSD indicating the deflection of the trapping laser beam (red) due to applied force. (**C**)Left: Schematic of a smFRET assay based on confocal imaging of a protein (purple) diffusing in solution. Here, changes in protein conformation are detected by monitoring the FRET efficiency (i.e. low or high FRET) between a donor and acceptor fluorophore that are covalently linked, respectively, to relevant structural domains within the protein. The FRET efficiency depends on the distance between the fluorophores. Right: Principle of FRET: When the distance between donor and acceptor is small, electronic excitation of the donor results in non-radiative energy transfer to the acceptor, followed by acceptor fluorescence (i.e. high FRET). (**D**) Left: Schematic illustration of an AFM set-up used for imaging biological samples, such as DNA. The sample is probed using a flexible cantilever connected to a small tip. Interactions between the tip and the surface are measured by directing a laser beam onto the rear face of the cantilever and detecting the position of the reflected light on a PSD (yellow). As the sample is scanned in the *x*,*y*-plane, the signal on the PSD is kept constant by adjusting the *z*-position of a piezo actuator (orange) using a feedback loop between the actuator and the PSD. Note that the actuator can be connected to either the cantilever or the surface. A three-dimensional image of the sample can be obtained by measuring the changes in actuator *z*-position. This can be used, for example, to directly visualize the topology of a negatively supercoiled DNA plasmid (right). Image adapted from Figure 1B of (155).

Over the past 20 years, a range of single-molecule techniques have been applied to study Type 1A topoisomerases, most notably magnetic tweezers (43,56,59,88,98,99,114–121), dual-trap optical tweezers (74), smFRET microscopy (122–124) and AFM (75). A major advantage of such techniques over ensemble methods is that population averaging is avoided. Therefore, single-molecule techniques often allow the identification of both rare activity events and subpopulations within multi-state distributions. In addition, by monitoring single enzymes interacting with DNA over time, dynamic and kinetic information can be directly obtained, which can be difficult and in many cases impossible to achieve using ensemble assays (125). The most commonly used single-molecule technique for the study of topoisomerases is magnetic tweezers, in large part due to its ability to control the DNA linking number accurately (40,42,43,126–128). In magnetic tweezers, single DNA molecules are tethered between the surface of a flow cell and a micro-sized paramagnetic bead (Figure 3A, left). This is usually achieved by labelling each end of the DNA with digoxigenin and biotin moieties, respectively, such that the DNA molecules can be attached specifically on one end to a surface coated with anti-digoxigenin and on the other end to streptavidin-coated beads (3,4,129). The flow cell is placed on an inverted microscope and illuminated from above, allowing the beads to be imaged with bright-field microscopy. A permanent magnet (placed above the sample) is then used to apply an attractive force to the beads. By changing the height of the magnet (via a motorized device), the strength of the magnetic field applied to the beads can be modulated, allowing precise tuning of the tension applied to the bead-tethered DNA molecules. Depending on the strength of the magnet and the experimental design (such as bead size and flow cell height), forces of between 0.001 and 100 pN can typically be applied (125,130,131).

The magnitude of the applied force can be calibrated based on the Brownian motion of the beads, extracted from analysis of the bright-field images (3,125,130,132). When a single magnet is positioned in a vertical configuration above the sample, forces can be applied while at the same time allowing free rotation of the beads (114,121,133). Conversely, when using a pair of magnets positioned horizontally above the sample, the beads cannot rotate freely, but can be controllably rotated by turning the magnets (3,4) (Figure 3A, left). When the tethered DNA molecules are torsionally constrained, the latter configuration allows the DNA linking number to be increased (Δ*Lk* >0) or decreased (Δ*Lk* <0) in a well-controlled manner, depending on the direction the magnet is turned. Here Δ*Lk* = *Lk* – *Lk_0_*, where *Lk*_0_ represents the linking number of relaxed DNA. The above feature allows the formation of positive or negative DNA supercoiling, respectively. Furthermore, by increasing the DNA concentration, it is possible to tether multiple surface-bound DNA molecules to a single bead. In this way, two DNA molecules can be entwined (braided) by rotating the magnets (and thus the bead) (59,119,134). The extension of the DNA molecule(s) at a given force can be determined from the diffraction pattern of the imaged beads, which varies as a function of bead height (Figure 3A, right) (131,132). As a result, topoisomerase-mediated changes in DNA topology can be studied with high precision and in real-time by monitoring the change in bead height (and therefore the DNA end-to-end length) at a given force.

In recent years, increasingly advanced magnetic tweezers assays have been constructed, which feature additional functionalities. For example, temperature-controlled set-ups have been shown to enable the temperature-dependent activity of reverse gyrases to be measured directly (88,114,121). Another important advance has been the combination of magnetic tweezers with total internal reflection fluorescence (TIRF) microscopy. By monitoring fluctuations in the fluorescence signal from fluorescently-labelled proteins or DNA using smFRET (13,135) or PIFE (98) (see later), protein binding and dynamics can be correlated with changes in DNA topology measured with magnetic tweezers. Using TIRF, it is also possible to image interactions of proteins along the length of DNA molecules within a magnetic tweezers assay. However, this is more technically challenging as the DNA molecules must be stretched parallel to the surface using side-pulling magnetic tweezers (8,136), which involves a more complex experimental design.

Similar to magnetic tweezers, optical tweezers enable mechanical manipulation of single DNA molecules tethered to micro-sized beads. (137,138). However, rather than using a magnetic field, optical tweezers exploit the fact that dielectric particles (such as polystyrene beads) can be trapped in the center of a tightly focused near-infrared (NIR) laser beam (139). Here, a DNA molecule is attached on one end to an optically-trapped bead and on the other end to either the surface of a flow cell (140–142), a micropipette (11) or a second optically-trapped bead (74,143–148). The latter configuration is referred to as dual-trap optical tweezers. Each of these assays offer distinct advantages, as discussed in detail elsewhere (138,149). We focus our discussion here on dual-trap optical tweezers, owing to its recent application to study Type 1A topoisomerases (74). In this configuration (Figure 3B, left), DNA is typically tethered to the beads via biotin-streptavidin linkages (using biotin-labelled DNA and streptavidin-coated beads) (143). The DNA extension can be varied by changing the distance between the two beads (using steerable mirrors or acousto-optic deflectors to control the positions of the laser beams). This, in turn, allows forces of >200 pN to be applied to the DNA molecule (144). The DNA extension can be determined by imaging the bead positions using bright-field microscopy (143). The corresponding force can be measured using back-focal plane imaging of small deflections in the trapping laser light (which are proportional to the magnitude of the force) on a position-sensitive detector (PSD) (Figure 3B, right) (143). In contrast to magnetic tweezers, optical tweezers act as an extension clamp, rather than a force clamp. However, experiments can also be performed under constant force (usually for forces ≥5 pN), by using a feedback loop in which the distance between the two beads is continuously adjusted to maintain a preset force (141,148).

One potential disadvantage of optical tweezers is that experiments often have a lower throughput in comparison to magnetic tweezers (where many single DNA molecules, each tethered to a bead, can be probed in parallel). In addition, in dual-trap optical tweezers, the beads cannot be easily rotated, which in turn restricts the ability to generate supercoiled DNA, although recent advances have started to address this (as discussed later in the outlook) (145,150). Despite these issues, dual-trap optical tweezers offer several advantages over magnetic tweezers for studying DNA-protein interactions. First, the technique is highly compatible with a wide range of fluorescence imaging modalities, including wide-field (74,143–146), confocal (142) and super-resolution imaging (147). This is because the DNA molecule is oriented perpendicular to the microscope objective, allowing straightforward imaging of interactions along the length of the DNA molecule. Combined optical tweezers and fluorescence imaging has been exploited extensively, for example, to probe sequence-dependent interactions of DNA-binding proteins and dyes (144,145), track protein translocation on DNA (145,147,151) and correlate protein binding with mechanical changes to the DNA (137,146,151,152). A second unique advantage of dual-trap optical tweezers is that the DNA is not fixed to an immovable surface, and as a result, by integrating this assay within a multi-channel flow cell, the DNA molecules can be moved between different solutions. This provides an efficient approach to probe the sequential recruitment of different proteins to DNA, and additionally facilitates fluorescence imaging of DNA–protein interactions with minimal fluorescence background (74,137).

smFRET is a single-molecule fluorescence microscopy technique based on the use of a donor and an acceptor fluorophore, in which the donor emission and acceptor excitation spectra overlap (Figure 3C). The fluorophores are covalently linked to defined positions within a DNA molecule and/or protein. For short distances (≤8 nm) between the two fluorophores, excitation of the donor will result in non-radiative energy transfer from the donor to the acceptor and concomitant fluorescence emission from the acceptor, rather than from the donor fluorophore (152,153). The FRET efficiency, i.e. the probability that the donor excited state energy is transferred to the acceptor, depends on the relative distance between the two fluorophores, and can thus be used to monitor changes in distances of up to ∼8 nm. The two most common smFRET approaches are based on either confocal microscopy of freely diffusing molecules in solution or TIRF microscopy of surface-tethered molecules (154). In recent years, confocal smFRET microscopy has been applied with great success to study the conformational changes associated with reverse gyrase activity (122–124).

Finally, AFM represents a powerful means to visualize the effect of topoisomerases on the overall topology of DNA (75), and can be used, for example, to detect the presence of plectonemes (Figure 3D, right) (75,155). In this technique, the sample of interest is deposited on a surface and probed with a small tip (typically <20 nm diameter) connected to a flexible cantilever. Interactions of the tip with the surface can be detected by directing a laser beam onto the rear face of the cantilever and monitoring the reflected light on a PSD (Figure 3D, left) (125). One of the most common AFM modalities used to image biological samples is Amplitude Modulation (156,157). Here, the cantilever is oscillated near its resonance frequency with a set amplitude. This amplitude is damped due to interactions between the tip and the sample, resulting in a change in the PSD signal. This effect can be exploited to generate a three-dimensional image of the sample by scanning the sample in the *x,y*-plane, while adjusting either the cantilever or the surface in the *z*-direction (using a piezo actuator) to maintain a constant cantilever amplitude. The corresponding changes in actuator *z*-position (reflecting the height profile of the sample) can be measured with sub-nanometer resolution.

## MECHANISMS OF SUPERCOIL RELAXATION

**Figure 4.**
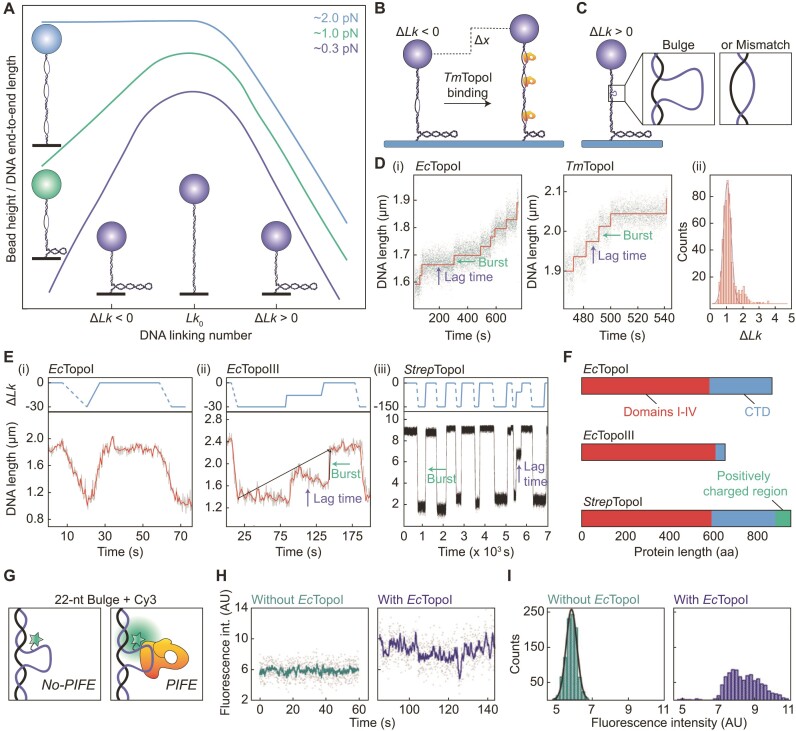
Overview of magnetic tweezers studies used to probe the mechanism of supercoil relaxation by bacterial Type 1A topoisomerases. (**A**) Schematic ‘hat-curve’ showing the measured DNA end-to-end length (in the absence of enzyme) as a function of bead rotations for three different tensions. Cartoons depict the DNA structure (either denatured, underwound or plectonemes) and show how the structure depends on both the direction of supercoiling and the applied force. The schematic is based on information provided in (15). (**B**) Schematic depicting how *Tm*TopoI can stabilize the formation of denatured, underwound DNA (at the expense of plectonemes) in negatively supercoiled DNA (Δ*Lk* <0) at intermediate (e.g. 0.9 pN) forces. (**C**) Schematic of a magnetic tweezers assay based on a positively supercoiled DNA molecule (Δ*Lk* >0) containing either a bulge or a mismatch. The bulge and the mismatch each enable the binding of a single topoisomerase enzyme to the DNA. (**D**) (i) Representative traces showing the change in DNA end-to-end length resulting from relaxation of positively supercoiled DNA containing a mismatch of 12 bp by *Ec*TopoI and *Tm*TopoI at 2 and 1.5 pN, respectively. The step-wise behaviour due to discrete bursts of activity separated by pauses (lag times) is indicated. (ii) Histogram of the measured burst sizes for *Ec*TopoI. A Gaussian fit reveals a mean burst size of 1.03 ± 0.1 *Lk*. Note that *Tm*TopoI displayed a similar burst size (43). Adapted from Figure 5 of (43) (Copyright (2002) National Academy of Sciences, U.S.A.). (**E**)Lower: Plots showing the decrease in the DNA end-to-end length due to the introduction of negative supercoiling (via magnet rotations) and subsequent increase in DNA end-to-end length due to relaxation of the negative supercoiling by (i) *Ec*TopoI (at 0.7 pN), (ii) *Ec*TopoIII (at 0.7 pN) and (iii) *Strep*TopoI (at 0.5 pN). Examples of bursts and lag times are indicated. Upper: Schematic depicting the change in *Lk* associated with data in the lower panels, where dashed and solid blue lines represent magnet-induced supercoiling, and enzyme-induced relaxation, respectively. Lower panels adapted from Figure 2A and D of (56) and Figure 5A of (120), respectively. Upper panels produced using the information provided in (56) and (120). (**F**) Comparison of the primary domains of *Ec*TopoI, *Ec*TopoIII and *Strep*TopoI, showing domains I-IV and the CTD (domain V) in red and blue, respectively. The positively charged region in the CTD of *Strep*TopoI containing multiple lysine repeats is shown in green. (**G**) Schematic representation of the use of PIFE to detect enzyme binding to a 22-nt bulge (containing a Cy3 dye) within positively supercoiled DNA in a magnetic tweezers assay. Upon binding, an increase in fluorescence intensity (i.e. PIFE) is observed. (**H**) Representative traces of Cy3 fluorescence intensity over time before (left) and after (right) addition of *Ec*TopoI, obtained using the assay described in panel G (at 1 pN). Adapted from Figure 2A and B of (98). (**I**) Histogram of the fluorescence intensities extracted from the traces shown in panel (H). Adapted from Figure 2A and B of (98).

Magnetic tweezers have been used to study supercoil relaxation by a wide range of taxonomically diverse Type 1A topoisomerases (43,56,88,98,115,116,118,120). These studies exploit the ability of magnetic tweezers to apply both torsional stress and tension to single molecules of DNA and have revealed a significant influence of the DNA tension on the ability of Type 1A topoisomerases to relax supercoils. This observation is, in part, due to the fact that negatively supercoiled DNA can adopt different structures depending on the applied tension (Figure 4A). At forces below ∼0.5 pN, negative torsional stress is primarily stored as plectonemes, whereas changes in twist are increasingly favoured over plectonemes as the tension increases (3–8,15). These changes in twist (underwound regions) often result in a denatured left-handed structure, known as L-DNA, with an average helicity of approximately -12 to -15 nucleotides (nt) per turn (11,14). However, strand-separated bubbles (5,16) and other left-handed forms (such as Z-DNA (13,17,18)) are also thought to occur, depending on the sequence, ionic strength and temperature. Since these underwound structures are more extended than plectonemes, the DNA end-to-end length of negatively supercoiled DNA increases as the tension is increased. At forces of ∼2 pN, negative torsional stress is fully absorbed through the formation of denatured, underwound DNA and the end-to-end length is similar to that of relaxed (i.e. non-supercoiled) DNA (Figure 4A) (3,4,6–9,15).

By measuring the change in DNA end-to-end length at constant force (through monitoring the bead height), magnetic tweezers studies have demonstrated that, on negatively supercoiled DNA, Type 1A topoisomerases show substantially lower relaxation activity at forces <0.5 pN than at ∼0.5 pN (43,56,116,118,120). This is due to the reduced abundance of denatured, underwound DNA at very low forces (3,4,6–9,15). In parallel, magnetic tweezers studies have additionally revealed that Type 1A topoisomerases can promote melting of negatively supercoiled DNA, independent of their catalytic activity. This was demonstrated, for example, by monitoring the DNA end-to-end length at a constant force of 0.9 pN using the catalytically inactive form of *Tm*TopoI (Figure 4B) (118). Although supercoil relaxation by catalytically active Type 1A topoisomerases is more efficient at intermediate forces (∼0.5–2 pN) than at low forces (<0.5 pN), this activity nonetheless decreases as the tension is increased further (despite the fact that denatured, underwound DNA is favoured over plectonemes at higher tensions) (43,118). For example, for *Ec*TopoI and *Tm*TopoI, efficient relaxation activity was observed on negatively supercoiled DNA at 0.53 pN, whereas no such activity was detected above 2.8 pN (43). This indicates that these enzymes act against the applied force during either DNA binding and/or strand passage. Furthermore, using magnetic tweezers, it was revealed that *Tm*TopoI and *Streptomyces coelicolor* TopoI fail to completely relax negatively supercoiled DNA, such that the final topological state generated is associated with a small supercoiling density (118,120). This latter observation is consistent with the first reported gel-electrophoresis experiments with *Ec*TopoI (31), and suggests that at very low supercoiling densities, the remaining torsional stress within the DNA molecule is too low to drive the catalytic mechanism.

Further insight into the mechanism of supercoil relaxation by Type 1A topoisomerases has been obtained from magnetic tweezers experiments using positively supercoiled DNA substrates. These substrates have two advantages over negatively supercoiled substrates. First, as was shown in gel-electrophoresis experiments (31,48), and subsequent magnetic tweezers studies (43,56,118), Type 1A topoisomerases cannot relax positively supercoiled substrates unless these contain a single-stranded region, such as a mismatch or a bulge, for the enzyme to bind to (Figure 4C). Thus, the presence of a small mismatch or bulge (<30 bp or nt, respectively) in positively supercoiled DNA can be exploited to ensure that only a single topoisomerase binding site is available. This is especially relevant for magnetic tweezers, as they have the resolution to detect the catalytic activity of a single enzyme in real-time. The ability to monitor single-enzyme activity is more difficult to achieve with negatively supercoiled DNA because, in this case, multiple ssDNA regions (and thus binding sites) often exist. The second advantage of positively supercoiled substrates is that they exhibit a (compact) plectonemic structure at forces up to at least 3 pN. This is in contrast to negatively supercoiled DNA, which is highly denatured at forces >1 pN and thus has a similar end-to-end length as non-supercoiled DNA at these elevated forces (Figure 4A) (15). As a result, by monitoring the change in end-to-end length of a positively supercoiled DNA substrate containing a mismatch or a bulge using magnetic tweezers, supercoil relaxation can be measured directly under rate-limiting tensions (e.g. 1–2 pN) (43). Under these conditions, the fundamental steps of the enzyme’s catalytic cycle can be detected, which is not possible using bulk biochemical methods. For example, using magnetic tweezers to manipulate a positively supercoiled substrate containing a 12-bp mismatch, it was demonstrated that, at ∼2 pN, *Ec*TopoI and *Tm*TopoI relax supercoils in discrete steps of exactly 1 *Lk*, separated by short periods of inactivity (lag times) (Figure 4D) (43). This provided the first direct evidence that these enzymes relax supercoiled DNA via an enzyme-bridged strand-passage mechanism, since a swivel mechanism (exhibited by Type 1B and Type 1C topoisomerases) would have resulted in an exponential distribution of step sizes (40,42,43). Moreover, the fact that this observation was made for evolutionarily distinct enzymes (i.e. *Ec*TopoI and *Tm*TopoI), and that a similar observation was later also made for *Ec*TopoIII (56), indicates that the enzyme-bridged strand-passage mechanism is conserved throughout all Type 1A topoisomerases.

Interestingly, even under rate-limiting conditions, steps larger than 1 *Lk* were occasionally observed for all three enzymes studied above. This indicates that these enzymes can sometimes resolve supercoils in bursts of multiple catalytic cycles (43,56). The processivity of supercoil relaxation (i.e. the number of *Lk* changes per burst) increased when the size of the mismatch was extended. For example, expanding the mismatch length from 12 to 27 bp increased the burst size from 1 to 1.5 *Lk* for *Ec*TopoI and from 1.5 to 3 *Lk* for *Ec*TopoIII. An even larger effect was observed for substrates containing a bulge, rather than a mismatch, with the processivity increasing 3–4 fold upon increasing the bulge size from 12 to 27 nt for both *Ec*TopoI and *Ec*TopoIII (56). These findings may be explained by steric effects: a longer region of mismatched DNA is expected to have a greater flexibility than a shorter region and a bulge is more flexible than a mismatch. The lag times between bursts also appear to be affected by the DNA structural flexibility. For example, for both *Ec*TopoI and *Ec*TopoIII, the lag times on positively supercoiled DNA containing a bulge decreased when the size of the bulge was increased. Moreover, the lag times for *Ec*TopoI were significantly reduced when the substrate contained a 27-nt bulge rather than a (less flexible) mismatch of the same size (56). Taken together, these findings demonstrate that DNA flexibility has a major influence on both the burst size and the lag time associated with supercoil relaxation by Type 1A topoisomerases. In support of this, it was observed that the rate of positive supercoil relaxation by both *Ec*TopoI and *Tm*TopoI decreased less as a function of tension when the substrate contained a 25-nt bulge, rather than a 12-bp mismatch (43,118). This is likely because the structural flexibility of a bulge is less affected by tension than that of a mismatch.

### Mechanistic basis for enzyme processivity and pause duration

Using magnetic tweezers, Terekhova *et al.* demonstrated that the overall rate of supercoil relaxation for *Ec*TopoIII is significantly lower than that for *Ec*TopoI (Figure 4E, cf. (i) and (ii)) (56), in agreement with earlier biochemical work (49,91). Moreover, by detecting the relaxation of supercoiling at the single-molecule level (using positively supercoiled DNA containing either a bulge or a mismatch), it was revealed that this difference is solely explained by a 4-fold longer lag time for *Ec*TopoIII compared with *Ec*TopoI (56). Interestingly, despite the overall reduced rate of supercoil relaxation in the case of *Ec*TopoIII, the processivity of *Ec*TopoIII was found to be higher than that of *Ec*TopoI: on average, *Ec*TopoIII exhibited 46% larger bursts than *Ec*TopoI (56). These differences have been attributed to a positively charged loop (called the decatenation loop) that is present in *Ec*TopoIII, but absent in *Ec*TopoI (Figure 2A) (91,158). This loop, which lies in close proximity to the gate, is important for supercoil relaxation, and essential for (de)catenation by *Ec*TopoIII (158). It has been postulated that the decatenation loop facilitates strand passage, likely by serving as an additional binding site for the T-segment (91,99,158).

The proposed role of the decatenation loop may explain the higher processivity associated with *Ec*TopoIII, but cannot explain its longer lag times. One possibility is that the difference in pausing behaviour between *Ec*TopoI and *Ec*TopoIII is due to the shorter CTD of *Ec*TopoIII (Figure 4F) (51,159). This would be consistent with the observation that *Ec*TopoI and *Tm*TopoI, which differ most notably in their CTDs (160), also show a substantial difference in the observed mean lag times (43). Various *in vitro* bulk studies have revealed that the CTD of *Ec*TopoI plays a role as an activator that stabilizes the core domain, and increases substrate recognition and DNA binding efficiency (51,115,159). These roles of the CTD may also explain why *Ec*TopoI relaxes positively supercoiled DNA containing a small mismatch (12 bp) in all cases, whereas relaxation of the same DNA construct by *Ec*TopoIII occurs in only 20% of cases (56). Nevertheless, whether there is a direct link between the CTD, substrate recognition and pause durations has yet to be resolved.

Further insight into the mechanistic basis for supercoil relaxation came recently from Gunn *et al.*, using a combination of magnetic tweezers and TIRF microscopy (98). These experiments utilized a positively supercoiled DNA molecule containing a 22-nt bulge labelled with the fluorescent dye Cy3. This dye displays a higher fluorescence intensity when a protein is nearby via a phenomenon known as protein-induced fluorescence enhancement (PIFE) (Figure 4G) (161,162). PIFE was used to detect the binding of *Ec*TopoI to the bulge, through an increase in the Cy3-fluorescence intensity (Figure 4H) (98). Moreover, by measuring the fluorescence intensity over time when the protein was bound (Figure 4H, right), small but significant fluctuations were observed, resulting in a skewed intensity distribution (Figure 4I). These fluctuations were attributed to conformational changes associated with the catalytic cycle of the enzyme. Such changes were only detected in the presence of magnesium (98), substantiating previous reports that magnesium is vital for the catalytic activity of Type 1A topoisomerases (103,105). When magnesium was present, and supercoil relaxation occurred, the Cy3-fluorescence intensity rarely returned to a no-PIFE state (98). This suggests that pauses between relaxation events are not a result of protein unbinding. Strikingly, the Cy3-fluorescence intensity remained fluctuating during each pause, indicating that *Ec*TopoI changed its conformation repeatedly without effectuating strand passage (98). It was proposed that these fluctuations represent failed attempts of the enzyme to capture the T-segment. These unsuccessful strand-capture events were hypothesized to be a consequence of suboptimal positioning of the enzyme on the DNA.

Unlike most bacterial species, actinobacteria possess only one Type 1A topoisomerase (115,120,163), known as both TopA and TopoI. In contrast to other bacterial TopoI enzymes, actinobacterial TopoI exhibits a much longer CTD, which includes a positively charged stretch containing multiple lysine repeats (Figure 4F), but lacks a zinc-binding motif (115,120,163). Magnetic tweezers studies of TopoI from the actinobacterium *S. coelicolor (Strep*TopoI) found that this enzyme relaxes negatively supercoiled DNA (at <1 pN) with a much higher processivity than either *Ec*TopoI or *Ec*TopoIII (Figure 4E) (115,120). In the majority of cases, relaxation of negatively supercoiled DNA by *Strep*TopoI was achieved in a single burst of up to 150 *Lk*, limited only by the number of turns introduced through bead rotation (120). This contrasts with mean bursts of 20 and 28 *Lk* reported for *Ec*TopoI and *Ec*TopoIII, respectively, on similar substrates (56).

In order to investigate whether the extreme processivity of *Strep*TopoI can be attributed to the C-terminal lysine repeats, magnetic tweezers were used to monitor supercoil relaxation by a series of mutant strains (115). These included (i) a mutant lacking the stretch of lysine repeats (*Strep*TopoI881) and (ii) a mutant in which the CTD is replaced by that of *Ec*TopoI (*Strep*TopoICTD*_Ec_*). In each case, a substantial reduction in the burst size was observed compared to the wild-type, with a 2-fold and 17-fold decrease in the number of ‘large bursts’ (defined as Δ*Lk* >75% of the initially introduced turns) for *Strep*TopoICTD*_Ec_* and *Strep*TopoI881, respectively. This demonstrates that both mutants relax negative supercoils with a significantly reduced processivity compared to the wild-type protein. Moreover, by measuring the time between enzyme addition and the first relaxation event (initial lag time), it was found that both mutants show a much longer initial lag time than the wild type. This was most significant in the case of *Strep*TopoI881, where the initial lag time was >20-fold longer, although the enzyme concentration was 100-fold higher. Furthermore, complementary surface-plasmon resonance experiments revealed that both mutants dissociate 3-fold more rapidly from DNA than the wild-type protein. On the basis of these results, it was concluded that the efficient supercoil relaxation exhibited by wild-type *Strep*TopoI is directly correlated with the presence of the C-terminal lysine repeats. The authors speculate that this positively charged tail might interact with negative charges in domain IV of the enzyme (which are not present in *Ec*TopoI or *Ec*TopoIII), and therefore stabilizes the enzyme–DNA complex (115).

## MECHANICS OF GATE OPENING

As discussed above, a range of structural (67,90–96), biochemical (36,48,96,97,102) and magnetic tweezers (43,98) studies support an enzyme-bridged strand-passage model as the mechanism for Type 1A topoisomerase activity. However, direct observation of gate opening has only recently been accomplished by probing the interaction of single Type 1A topoisomerases with ssDNA (99). In this study, Mills *et al.* monitored the change in end-to-end length of ssDNA in the presence of *Ec*TopoI or *Ec*TopoIII, using magnetic tweezers. To this end, a DNA-hairpin structure was first unfolded (at forces of ∼22–24 pN) to yield a stretch of 1074 nt of ssDNA. In the presence of either enzyme, a step-wise increase in the ssDNA length was observed. Such elongation was absent when the non-cutting mutant of *Ec*TopoI (Y319F) was used, indicating that the elongation corresponds to ssDNA gate opening. By measuring the stepwise length increase upon successive enzyme binding to the unfolded hairpin, the mean size of the open gate was determined to be ∼6.6 nm for both *Ec*TopoI and *Ec*TopoIII (99). A comparable gate size was also reported recently for *Sc*TopoIII (∼8.6 nm), using a similar magnetic tweezers approach (117). The measured sizes of the open gates for these enzymes are relatively large considering that a gap in ssDNA of only ∼1 and ∼2 nm is required for passage of ssDNA and dsDNA, respectively. However, complementary molecular dynamics simulations revealed that, upon gate opening, the distance between domains III and IV of the enzyme (Figure 2C) is much smaller than the opening created in the cleaved ssDNA. These simulations predicted that the ssDNA opening must exceed 3.8 or 5.7 nm to create an enzyme-bridged gate large enough to transfer a segment of ssDNA or dsDNA, respectively (99).

**Figure 5.**
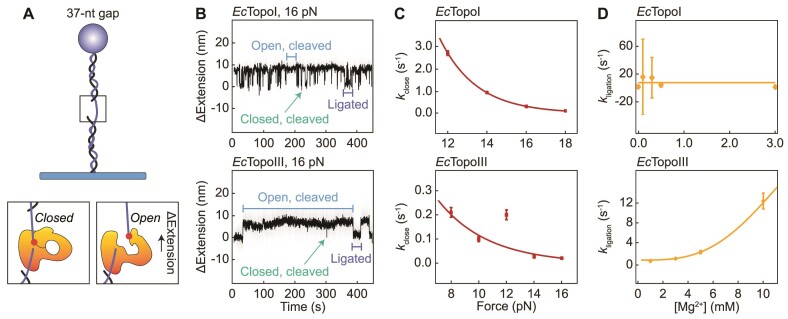
Gate dynamics of *Ec*TopoI and *Ec*TopoIII probed using magnetic tweezers. (**A**)Upper: Schematic of a gapped DNA substrate used by Mills *et al.* (99) to directly measure the gate opening dynamics of *Ec*TopoI and *Ec*TopoIII, respectively. Lower: Schematic of the enzyme bound to the ssDNA gap in a closed, cleaved and open, cleaved conformation, respectively. The latter conformation results in an increase in the DNA end-to-end length (ΔExtension). (**B**) Representative extension-time traces for the substrate described in panel A at 16 pN in the presence of *Ec*TopoI (upper) and *Ec*TopoIII (lower). The ligated state (purple), closed, cleaved state (green) and open, cleaved state (blue) are indicated. (**C**) Plots showing the closing rates (*k*_close_) for *Ec*TopoI (upper) and *Ec*TopoIII (lower) as a function of the applied force. An exponential decay function was fitted to the data (red lines), which yielded the lifetime of the open, cleaved state (1/*k*_close_) in the absence of force. (**D**) Plots showing the influence of the magnesium concentration ([Mg^2+^]) on the re-ligation rates (*k*_ligation_) for *Ec*TopoI (upper) and *Ec*TopoIII (lower). A linear function was fitted to the data for *Ec*TopoI and an exponential growth function was fitted to the data for *Ec*TopoIII (yellow lines). Panels B, C and D adapted from Figures 3B, 4C and D of (99), respectively.

To probe the kinetics of gate opening, Mills *et al.* used a DNA substrate containing a single-stranded gap of 37 nt that can accommodate the binding of a single enzyme (Figure 5A) (99). Once the enzyme was bound to the short ssDNA region, opening and closing of the enzyme-bridged gate was observed as a transient change in the DNA extension at a constant tension (Figure 5B). Independent of the applied force (8–18 pN), open-gate sizes were measured to be ∼5.9 and ∼5.5 nm for *Ec*TopoI and *Ec*TopoIII, respectively. This is broadly in agreement with the open-gate sizes obtained using the unfolded hairpin assay and the molecular dynamics simulations reported above. Using an unbounded hidden-Markov model to describe the kinetic data, it was found that the gate dynamics of both *Ec*TopoI and *Ec*TopoIII could be explained by a three-state model in which the protein–ssDNA complex can exist in either a ligated state, a short-lived closed, cleaved state, or an open, cleaved state (Figure 5B) (99).


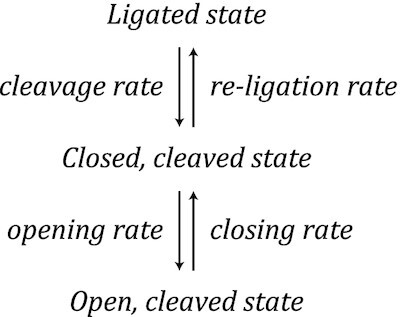


For both enzymes studied, histograms of the measured lifetimes of each state were obtained and an exponential function was fitted to the data to determine the cleavage, opening and closing rates. The re-ligation rate was estimated by considering the kinetic competition between opening and re-ligation from the closed, cleaved state. By determining the reaction rates at different forces, it was revealed that the cleavage, opening and re-ligation rates were insensitive to the applied force for both *Ec*TopoI and *Ec*TopoIII. Since mechanical opening of the gate was, nonetheless, expected to be force sensitive, Mills *et al.* postulated that the apparent absence of force dependence indicates that gate opening involves a rate-limiting step prior to mechanical opening (99).

The closing rate, and thus the lifetime of the open state, was, however, dependent on the applied force for both enzymes (Figure 5C). Using an exponential function to describe the change in closing rate as a function of force, Mills *et al.* revealed that the lifetime of the open state in the absence of force is several orders of magnitude longer for *Ec*TopoIII than for *Ec*TopoI (99). Since *Ec*TopoIII is more efficient at decatenating DNA than *Ec*TopoI (49,59,60,158), it was suggested that an increased lifetime of the open state in the case of *Ec*TopoIII may facilitate the capture of a distant T-segment (99). Interestingly, the presence of magnesium affected the gate dynamics of *Ec*TopoI and *Ec*TopoIII differently, with the re-ligation rate depending strongly on the magnesium concentration only in the case of *Ec*TopoIII (Figure 5D). It was hypothesized that this difference may be explained by the presence of a lysine residue (K8) in the TOPRIM domain of *Ec*TopoIII, which is absent in *Ec*TopoI. This lysine residue possibly results in a weaker binding of magnesium to *Ec*TopoIII compared to *Ec*TopoI (99).

## INTER-STRAND EXCHANGE

**Figure 6.**
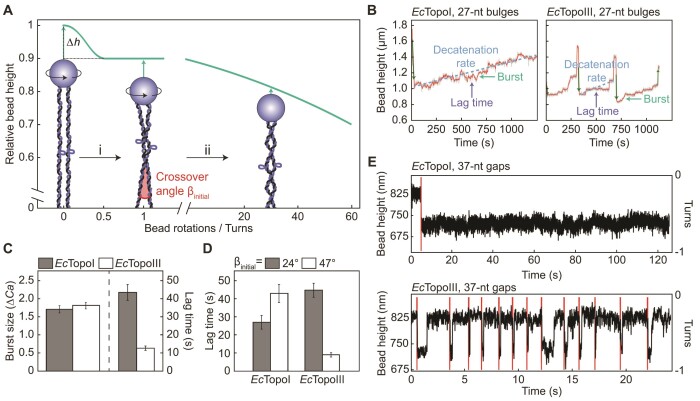
Decatenation activity of *Ec*TopoI and *Ec*TopoIII probed using magnetic tweezers. (**A**) Schematic showing the relative extension of two parallel DNA molecules tethered between a single magnetic bead and the surface at constant tension as a function of magnet turns. The sharp decrease in bead height upon a half turn of the bead is highlighted as Δ*h*. While only one direction of magnet rotations (positive) is shown here, a similar behaviour is observed when applying negative turns of the magnet. The schematic is based on the information provided in (134). Cartoons depict the substrates used by Terekhova *et al.* (59) to study the decatenation activity of *Ec*TopoI and *Ec*TopoIII. Here, two DNA molecules, each containing a 27-nt bulge, are entwined (braided) by rotating the magnetic bead by up to 30 to 35 turns. The initial crossover angle (β_initial_) is indicated. The green arrows highlight the change in bead height associated with each substrate. (**B**) Representative traces showing the change in bead height during decatenation by *Ec*TopoI (left) and *Ec*TopoIII (right) using the assay described in panel A (for a 30–35-turn braid) at 2 pN. The traces exhibit step-like behaviour, corresponding to bursts of activity separated by pauses (lag times) that together determine the decatenation rate, as indicated. Dark green vertical arrows denote changes in bead height due to magnet-induced rotations of the bead. Adapted from Figure 1F and I of (59). (**C**) Bar plot showing the mean burst size and lag time for *Ec*TopoI (grey) and *Ec*TopoIII (white), respectively, during decatenation of a 30–35-turn braid at 2 pN. The burst sizes are defined in units of Δ*Ca*, where *Ca* is the number of catenanes (i.e. turns) removed. Adapted from Figures 2A and 3A, respectively, of (59). (**D**) Bar plot showing the effect of the magnitude of β_initial_ on the mean lag time for *Ec*TopoI and *Ec*TopoIII measured during decatenation of a 30–35-turn braid at 2 pN. Grey and white bars represent small (24°) and large (47°) angles, respectively. Adapted from Figure 3B of (59). (**E**) Change in bead height as a function of time before and after braiding two DNA molecules, each containing a gap of 37 nt, by one turn in the presence of *Ec*TopoI (upper) and *Ec*TopoIII (lower). The red lines indicate magnet-induced rotations of the bead. Adapted from Supplementary Figure S3A and C of (119).

Since two DNA molecules (tethered between a single bead and a surface) can be entwined using magnetic tweezers, this approach is well-suited to measure the decatenation activity of Type 1A topoisomerases (59,119). As Figure 6A highlights, for two DNA molecules that are aligned in parallel, there is a marked reduction in bead height (Δ*h*) upon a half turn of the bead (corresponding to the first crossover). Further rotation of the bead leads to a more gradual decrease in bead height as the number of crossovers increases. Thus, by monitoring the change in bead height at a given force, the rate of decatenation can be determined directly. Terekhova *et al.* used this approach to study the decatenation mechanisms of *Ec*TopoI and *Ec*TopoIII on two entwined dsDNA molecules that were braided 30–35 times around one another (59). Using intact dsDNA, no decatenation was detected, while negligible activity was reported for dsDNA containing nicks in the backbone. In contrast, both *Ec*TopoI and *Ec*TopoIII were found to decatenate dsDNA molecules containing a 27-nt bulge, confirming that (de)catenation by Type 1A topoisomerases requires ssDNA (49). Importantly, these magnetic tweezers experiments revealed that the rate of decatenation by *Ec*TopoIII is 2-fold higher than by *Ec*TopoI (59). This chimes with earlier biochemical studies (49,60,158), and is thought to be due to the presence of the decatenation loop in *Ec*TopoIII (Figure 2A).

In addition to measuring the overall rate of decatenation, the use of magnetic tweezers has shed unique insight into the underlying mechanism, owing to the fact that the processivity of single enzymes can be measured directly. In this way, Terekhova *et al.* revealed that decatenation by both *Ec*TopoI and *Ec*TopoIII is processive and occurs in bursts of activity separated by pauses (Figure 6B), mirroring the behaviour observed during supercoil relaxation by these enzymes (Figure 4E). By measuring the burst sizes and pause durations, it was discovered that the higher decatenation rate of *Ec*TopoIII, relative to *Ec*TopoI, can be explained solely by ∼3.4-fold shorter lag times in the case of *Ec*TopoIII (Figure 6C) (59). In the same study, Terekhova *et al.* also investigated how the geometry of the braid influences the decatenation activity. The geometry of each braid can be described by the angle between the two entwined DNA molecules, termed the crossover angle, β (Figure 6A). β depends on a range of factors, including the separation between the two DNA molecules prior to braiding and the number of entwined turns (59,134). Terekhova *et al.* used a simplified model to estimate the initial crossover angle, β_initial_, of each braid, based purely on the difference in bead height between 0 and 1 rotations (defined here as Δ*h_0__–__1_*) and the extension of the DNA after one turn (*l*). Using the equation β_initial_ = 2arccos((*l*−Δ*h_0__–__1_*)/*l*), the authors divided their DNA braids into two groups, with either a small or large β_initial_ (with average values of ∼24° or ∼47°, respectively) (59). In this way, it was demonstrated that the pause durations between bursts were affected by β in contrasting ways for *Ec*TopoI and *Ec*TopoIII, respectively. For *Ec*TopoI, large β braids resulted in longer lag times (1.6-fold) than smaller β braids. In contrast, for *Ec*TopoIII, pause durations were reduced by up to 5-fold for larger β compared with smaller β (Figure 6D). These observations reveal that *Ec*TopoIII decatenation activity is stimulated by a larger crossover angle, raising the possibility that TopoIII enzymes may have evolved to resolve structures (such as (hemi)catenanes) that are under tension during mitosis and meiosis (59).

In a separate magnetic tweezers study, two dsDNA molecules, each containing a 37-nt gap, were entwined by one bead rotation in order to monitor decatenation by *Ec*TopoI and *Ec*TopoIII, respectively. Interestingly, in contrast to the results described above, *Ec*TopoI was unable to decatenate the DNA molecules, even when using a 30-fold higher protein concentration than was used to detect decatenation by *Ec*TopoIII (119) (Figure 6E). The difference in the results between these two studies may be explained by the higher structural flexibility of a bulge compared to a gap and/or the lower torsional stress introduced by a single-turn braid compared to a 30–35-turn braid. This would be consistent with the observation that Type 1A topoisomerases exhibit a higher rate of supercoil relaxation on a bulge compared to a mismatch (56) and a reduced rate of relaxation of negatively supercoiled DNA with a low supercoiling density (31,118,120).

## INTERPLAY WITH PROTEIN BINDING PARTNERS

**Figure 7.**
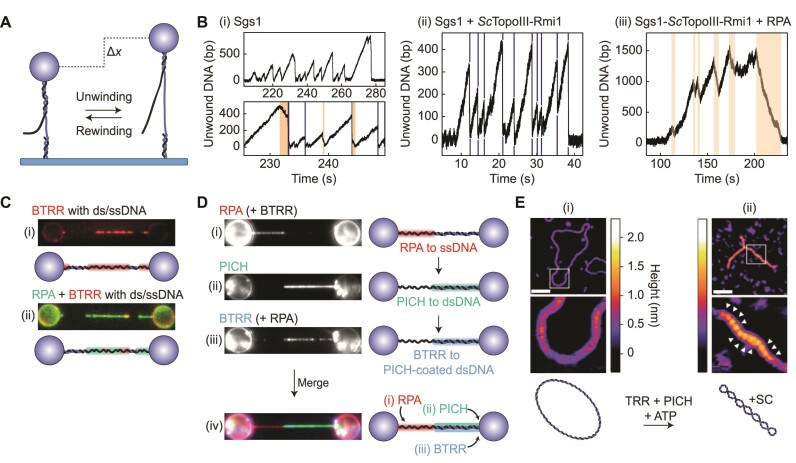
The interplay of eukaryotic Type 1A topoisomerases with partner proteins, as determined using magnetic tweezers, optical tweezers and AFM. (**A**) Schematic of a magnetic tweezers assay used by Kasaciunaite *et al.* (117) to probe the unwinding activity of Sgs1. Here, the DNA substrate contains a gap of 38 nt, along with a 40-nt ssDNA ‘flap’. In the presence of ATP, duplex unwinding by Sgs1 (initiated at the ds/ssDNA junction) will result in an increase in the DNA end-to-end length (Δ*x*), which will be reversed upon re-winding. (**B**) Representative traces showing the change in the number of unwound bp (derived from changes in the DNA end-to-end length) for the substrate shown in panel A in the presence of (i) Sgs1, (ii) Sgs1 and *Sc*TopoIII-Rmi1 and (iii) Sgs1, *Sc*TopoIII-Rmi1 and yeast RPA (at forces in the range of 10–35 pN). Note that ATP was present in all cases. Gradual rewinding events are highlighted in orange shading, while rapid renaturation events are indicated by dark blue lines. Adapted from Figures 1B, 4B and E of (117), respectively. (**C**) Representative fluorescence images and corresponding schematics for a DNA molecule containing mixed ds/ss regions (tethered between optically-trapped beads) after incubation with (i) human BTRR (red) or (ii) human BTRR and RPA (red and green, respectively). BLM and RPA were labelled with mCherry and mStrawberry, respectively. Images adapted from Supplementary Figure S9B of (74). (**D**) Representative fluorescence images (left) and corresponding schematics (right) for DNA substrates containing mixed ds/ss regions (tethered between optically-trapped beads) after sequential incubation in channels containing: (i) human RPA-mStrawberry (red) and BTRR (containing BLM-mCherry); (ii) human PICH-eGFP (green); and (iii) human RPA-mStrawberry and BTRR (containing BLM-mCherry, blue). Note that in (i) and (iii) BTRR is excluded from the ssDNA by RPA. The final image and schematic (iv) show a composite of the above results. Images adapted from Figure 5B of (74). (**E**) Representative AFM images and corresponding schematics of a relaxed DNA plasmid prior to (i) and after (ii) incubation with human PICH and TRR (in the presence of ATP). The white arrows in (ii) indicate the locations of increased DNA height due to crossovers of the double helix (i.e. writhe). The measured height is indicated by the colored scale bar. The white scale bar represents 100 nm. Images adapted from Figure 2C of (75).

The ability of TopoIII enzymes to resolve DNA entanglements *in vivo* is often mediated through interactions with other enzymes. One of the most studied examples is the association of a TopoIII enzyme with a RecQ-family helicase, resulting in the dissolvasome complex (72). In concert with other ssDNA binding proteins, the dissolvasome plays a vital role in resolving late-replication and recombination intermediates (28,62,63). Biochemical studies have shown that formation of the dissolvasome stimulates both the decatenation activity of the constituent TopoIII enzymes and the DNA unwinding activity of the corresponding RecQ-family helicases (24,25,63,70,164–166). However, the mechanisms underlying these effects have proved elusive. In recent years, single-molecule assays have shed new light on the molecular interactions of the dissolvasome. For instance, Kasaciunaite *et al.* used magnetic tweezers to probe the yeast dissolvasome (consisting of *Sc*TopoIII-Rmi1 and the RecQ-helicase Sgs1) and its interaction with the yeast ssDNA binding protein RPA (117). This study employed a dsDNA substrate containing a 38-nt ssDNA gap along with a 40-nt ssDNA ‘flap’, providing a ds/ssDNA junction that facilitates initiation of DNA unwinding by Sgs1 (Figure 7A). By monitoring the DNA end-to-end length under constant tension (15–25 pN) in the presence of ATP, the unwinding rate of Sgs1 was measured to be 65 bp·s^–1^. Sgs1 unwinding activity was typically terminated by either strand switching, which resulted in short rewinding events (24% of cases), or rapid renaturation of the DNA (Figure 7B, (i)). Rapid renaturation is likely due to a reduced ability of Sgs1 to maintain contact with both strands of the DNA, which allows the ds/ssDNA junction to push back the helicase. In the presence of *Sc*TopoIII-Rmi1 (Figure 7B, (ii)), the rate of DNA unwinding by Sgs1 increased by 32% and the frequency of short rewinding events decreased from 24% to 5% (117). These results reveal that *Sc*TopoIII-Rmi1 promotes unwinding and inhibits strand switching by Sgs1, which substantiates the proposed role of TopoIII-Rmi1/RMI1 during branch migration of a double Holliday junction (23,24,27,28).

Consistent with biochemical studies (63,166), the magnetic tweezers measurements conducted by Kasaciunaite *et al.* demonstrated that the unwinding activity of Sgs1 also increased significantly in the presence of RPA (117). Moreover, the ability to probe the unwinding activity of Sgs1 directly at the single-molecule level revealed that the stimulated unwinding activity of Sgs1 by RPA is explained by an approximately 2-fold increase in processivity (i.e. the number of bp unwound before switching direction), rather than an increase in the unwinding rate. In fact, RPA reduced the unwinding rate of Sgs1 by 22%. Both the processivity and the unwinding rate of the Sgs1–*Sc*TopoIII–Rmi1 complex were similarly affected by the presence of RPA. Furthermore, rapid renaturation events (due to a loss of Sgs1 contact with both strands of the DNA) did not occur in the presence of RPA. As a result, unwinding events were exclusively terminated by gradual DNA rewinding by Sgs1 (Figure 7B, (iii)). This indicates that RPA promotes the interaction of Sgs1 with both strands of the DNA. Notably, the above effects were not observed when an Sgs1 mutant lacking the RPA binding site (167) was used, revealing that the stimulated unwinding activity of the dissolvasome by RPA is modulated by a physical interaction between Sgs1 and RPA (117).

The dissolvasome of higher eukaryotes consists of the helicase BLM and the topoisomerase complex TRR (TopoIIIα, RMI1 and RMI2) and is often referred to as BTRR. Using combined dual-trap optical tweezers and fluorescence imaging, Sarlós *et al.* visualized the interactions between fluorescently-labeled human BLM, TRR and RPA on DNA substrates containing both ds- and ssDNA (ds/ssDNA) (74). To this end, a multi-channel microfluidic flow cell was employed that facilitates sequential incubation of the DNA molecules in distinct channels containing BLM, TRR and RPA, either separately or combined. In this way, it was confirmed that BLM and TRR exhibit negligible binding to dsDNA. In contrast, BLM and TRR were observed to bind strongly to, and co-localize on, ssDNA (Figure 7C, (i)), independent of the order of incubation. This indicates that these proteins form a stable complex (BTRR) on ssDNA. Strikingly, when the DNA molecule was incubated in a solution containing both BTRR and RPA, almost no BTRR binding to ssDNA was observed, suggesting that BTRR is excluded from ssDNA by RPA (Figure 7C, (ii)) (74). A similar finding was later observed for *Sc*TopoIII by Kasaciunaite *et al.* (117), using the magnetic tweezers assay discussed above. In the latter study, ssDNA gate opening by *Sc*TopoIII (detected through sudden length increases of the DNA substrate at a constant force) was largely inhibited by RPA. Taken together, these results suggest that, *in vivo*, RPA may protect regions of ssDNA from damage due to undesired cleavage by TopoIII (74,117).

As well as its role in double Holliday junction resolution, BTRR is thought to facilitate resolution of ultra-fine DNA bridges (UFBs). These entangled DNA structures, present during anaphase, arise from late-replication intermediates and often involve a hemicatenane structure (168). *In vivo* immunostaining has previously revealed that the dsDNA regions of UFBs in both chicken and human cells are coated with both BTRR and the ATP-dependent dsDNA translocase PICH (169–171). In order to understand how BTRR interacts with PICH in the context of UFB resolution, Sarlós *et al.* extended their single-molecule approach (see above) by incubating ds/ssDNA molecules with fluorescently-labeled human RPA, PICH and BTRR in different orders (74). This revealed that, although BTRR is excluded from ssDNA by RPA, it is recruited to dsDNA by PICH (Figure 7D). Since recruitment of BTRR by PICH resulted in the presence of BTRR at ds/ssDNA junctions, it was proposed that this could facilitate initiation of unwinding by BLM and/or decatenation by TRR, ultimately leading to UFB resolution. Complementary biochemical studies showed that both PICH and RPA enhance the ability of BTRR to resolve late-replication intermediates. Together, these results suggest dual roles for PICH and RPA in both stimulation and recruitment of the dissolvasome to late-replication intermediates, such as UFBs (74).

It has additionally been shown that human PICH and TRR cooperate on dsDNA, in the presence of ATP, to induce positive supercoiling. This was demonstrated using 2D-gel electrophoresis experiments (which indicated that >10 positive supercoils are generated per 2.7 kb) and directly visualized by Amplitude Modulation AFM (75). The AFM images show a high level of compaction of torsionally constrained dsDNA following incubation with PICH and TRR (along with ATP), consistent with the formation of plectonemes (Figure 7E). Complementary magnetic tweezers experiments revealed that ATP-dependent translocation of PICH on torsionally constrained dsDNA extrudes loops of negatively supercoiled DNA, resulting in positively supercoiled domains adjacent to the loops (75). On the basis of these observations, it was proposed that TRR can relax the negatively supercoiled loops, while the positive supercoils are retained. Consistent with this model, PICH was also found to induce positive supercoils in the presence of other Type 1A topoisomerases, including *Ec*TopoI and *Ec*TopoIII, but not Type 1B or Type 2 topoisomerases (which relax both positive and negative supercoils) (75). The ability of PICH and TRR to work in concert to generate positively supercoiled DNA is reminiscent of reverse gyrase activity in (hyper)thermophilic organisms. It has been proposed that positive supercoiling may facilitate the resolution of UFBs by stimulating the activity of another topoisomerase, TopoIIα. The latter is a Type 2 topoisomerase and has been reported to exhibit enhanced decatenation activity on positively supercoiled catenanes (75,171,172).

## REVERSE GYRASES

Notwithstanding the cooperative interaction of TRR and PICH, reverse gyrases (RGs) are the only Type 1A topoisomerases that can generate positive DNA supercoiling. This feature is driven by the coupling of a Type 1A topoisomerase domain and an ATP-dependent helicase domain (85–88). The RG helicase domain consists of two RecA-like subunits, H1 and H2 (Figure 2B), which interact with DNA and bind and hydrolyze ATP (76). Crystal structures have revealed that H1 and H2 can together form either an open or closed conformation and it has been postulated that this conformational change allows for the generation of positive supercoiling (79,80). The presence of a small folding unit in H2, known as ‘the latch’ (Figure 2B) is essential for the positive supercoiling activity of RGs (173,174). The latch consists of two functional domains, a highly conserved β-hairpin and a less conserved globular domain (175). The latch is thought to transiently interact with the topoisomerase domain during the catalytic cycle and to contribute to DNA binding (79,80,124,175).

### Helicase conformational changes

**Figure 8.**
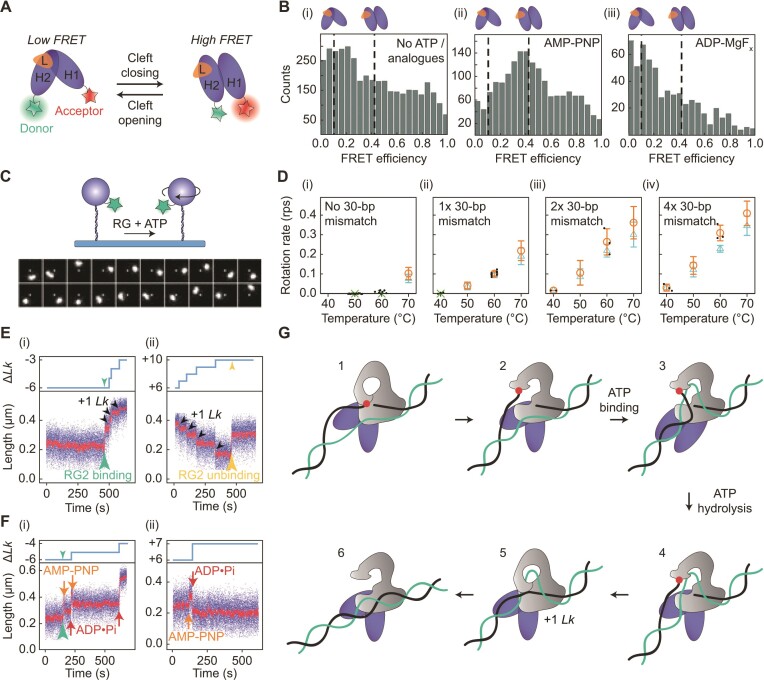
Overview of single-molecule studies of RG activity. (**A**) Schematic of a smFRET assay used by Del Toro Duany *et al.* (122–124) to study conformational changes of the helicase domain of RGs. Donor and acceptor dyes are covalently linked to H2 and H1 subunits, respectively. The FRET efficiency will increase upon closure of the cleft between H1 and H2. (**B**) Histograms of the measured FRET efficiencies (obtained using the assay described in panel A) based on the helicase domain of *Tm*RG in (i) the absence of ATP (or ATP-analogues), (ii) the presence of AMP-PNP and (iii) the presence of ADP-MgF_x_. In all cases shown, a ds/ssDNA substrate was present. Adapted from Figure 6C of (122) with permission from the PCCP Owner Societies. (**C**)Upper: Schematic of the assay employed by Ogawa *et al.* (114,121) to measure DNA overwinding by *St*RG using fluorescently-labelled magnetic beads that can freely rotate. Lower: Sample fluorescence images showing clockwise rotation of fluorescently-labelled beads, obtained using the assay shown above (corresponding to DNA overwinding by *St*RG at 0.5 pN and 71°C, in the presence of ATP). Lower panel adapted from Figure 2A of (121). (**D**) Plot showing the mean of the maximal overwinding rates (orange circles) and of the average overwinding rates (blue triangles) of *St*RG as a function of temperature for DNA substrates containing zero, one, two and four 30-bp mismatches, respectively, determined using the assay described in panel C (121). Black dots represent individual maximal rates, whereas green crosses highlight conditions where the enzyme was unable to overwind the DNA. Adapted from Figure 4 of (114). (**E**) Interaction of *Sso*RG2 with (i) negatively supercoiled and (ii) positively supercoiled DNA, respectively, in low ATP concentrations at 45°C and 0.2 pN, studied using a magnetic tweezers assay similar to that shown in Figure 3A. Lower: Changes in the DNA end-to-end length as a function of time. *Sso*RG2 binding and unbinding from the DNA are indicated by green and yellow arrowheads, respectively. Successive strand passage events (+1 *Lk*) are indicated by black arrowheads. Adapted from Figure 1C and E of (88). Upper: Schematics depicting the change in *Lk* associated with the data in the lower panels (produced using the information provided in (88)). (**F**) Interaction of *Sso*RG2 with (i) negatively supercoiled and (ii) positively supercoiled DNA, respectively, in the presence of both AMP-PNP and ATP at 45°C and 0.2 pN, studied using a similar assay as in panel (E). Lower: Changes in DNA end-to-end length as a function of time. Orange and red arrows indicate AMP-PNP binding and subsequent ATP hydrolysis (i.e. conversion from ATP to ADP•Pi), respectively. Adapted from Figure 4A and C of (88). Upper: Schematics depicting the change in *Lk* associated with the data in the lower panels (produced using the information provided in (88)). (**G**) Proposed model for supercoil generation by RGs, based on the findings from single-molecule studies. 1. RG binds to (or, in the case of *Sso*RG2, forms) a strand-separated bubble, allowing the topoisomerase domain (grey) to cleave one strand (the G-segment, black), resulting in the formation of an enzyme-bridged ssDNA gate. 2. The enzyme undergoes a conformational change, leading to gate opening. 3. Upon ATP binding, the helicase domain (purple) switches to a closed conformation, which induces partial rewinding of the bubble. This facilitates T-segment (green) binding to the central cavity of the open topoisomerase domain. 4. Subsequent ATP hydrolysis results in opening of the helicase cleft and re-formation of the bubble. 5. The enzyme-bridged ssDNA gate closes, followed by re-ligation of the G-segment, resulting in a change of +1 *Lk*. 6. The topoisomerase domain adopts an open conformation in order to release the T-segment from the cavity. Note that this catalytic cycle is also relevant for the (ATP-dependent) strand-passage mechanism during negative supercoil relaxation for *Sso*RG2.

In recent years, smFRET has been exploited to probe how the conformational changes of the helicase domain in RGs are correlated with both DNA binding and ATP hydrolysis. Del Toro Duany *et al.* studied the helicase domain of *Tm*RG in the presence of different DNA substrates, including ssDNA, dsDNA and ds/ssDNA junctions, by monitoring the FRET efficiency between a donor and an acceptor dye linked, respectively, to either H1 or H2 (Figure 8A) (122–124). Intermediate states in the ATP hydrolysis cycle were captured using the ATP analogues AMP-PNP (to mimic the binding of ATP, without subsequent hydrolysis), ADP-BeF_x_ (to mimic the pre-hydrolysed state of ATP), ADP-MgF_x_ (to mimic the state immediately after hydrolysis, but before phosphate release) and ADP (to mimic the state directly after phosphate release). In the absence of ATP analogues, the helicase domain adopted an open conformation (Figure 8B, (i)) (122), with a distance between the donor and acceptor dye of ∼7.5 nm (123). The FRET efficiency increased upon AMP-PNP binding, indicating that the cleft between H1 and H2 closes upon ATP binding (Figure 8B, cf. (i) and (ii)). The cleft remained closed in the presence of ADP-BeF_x_, but opened upon binding of ADP-MgF_x_, indicating that the helicase re-adopts the open state upon ATP hydrolysis (Figure 8B, cf. (ii) and (iii)). Release of the phosphate did not induce further conformational changes: the cleft between H1 and H2 remained open in the presence of ADP. The above observations were similar for all DNA substrates studied. However, the conformational change to the closed state in the presence of AMP-PNP was not observed in the absence of DNA (122). Taken together, these results demonstrate that the helicase domain functions as a DNA- and ATP-dependent switch that alternates between an open and closed conformation (122). Moreover, complementary fluorescence equilibrium DNA titration studies indicated that AMP-PNP and ADP-BeF_x_ binding (and thus the closed conformation of the helicase) is associated with an increased affinity for dsDNA (122,176). This has been proposed to enable positive supercoil generation by imposing a structural change in the DNA adjacent to the topoisomerase–ssDNA complex (76).

By monitoring the FRET efficiency within a helicase fragment from *Tm*RG that lacks the latch (Δlatch), Del Toro Duany *et al.* revealed that this truncated helicase domain does not adopt a closed conformation in the presence of both DNA and AMP-PNP (124). Moreover, although closing events could be observed at a later stage of the ATP-bound state (captured via ADP-BeF_x_), this occurred with a much lower efficiency compared to the wild-type (122,124). These results can be explained by a weaker affinity of the helicase domain for DNA in the absence of the latch. This hypothesis was supported by additional fluorescence equilibrium titration experiments. Interestingly, these latter experiments demonstrated that the difference in DNA affinity between the truncated and wild-type helicase domains was most stark upon ATP hydrolysis, particularly for the case of ssDNA (124). Together with the requirement of the (β-hairpin of the) latch for introduction of positive supercoiling (173,175), the above results suggest that the latch is responsible for guiding the single-stranded T- or G-segment upon ATP hydrolysis. It remains unclear, however, which of these two segments are in contact with the latch (76).

### Coupling between the helicase and topoisomerase domains

In order to gain mechanistic insights into how the conformational changes of the helicase domain are coupled to strand passage, magnetic tweezers studies have recently been performed on two different RGs, *Sulfolobus tokodaii* (*St*)RG (114,121) and *S. solfataricus* (*Sso*)RG2 (88). The former requires ATP only for DNA overwinding, whereas the latter requires ATP for both relaxation of negative supercoiling and DNA overwinding. Ogawa *et al.* monitored the overwinding activity of *St*RG on torsionally constrained DNA in two ways (114,121). In the first approach, the ability of *St*RG to generate positive supercoiling was investigated using a magnetic tweezers assay similar to that depicted in Figure 3A. In this assay, DNA overwinding (at forces < ∼3 pN) results in plectoneme formation, and thus supercoiling can be detected (at a given force) from a reduction in the DNA end-to-end length (Figure 4A). By performing these experiments at different forces, the rate of supercoiling by *St*RG at 71°C was found to decrease as a function of tension (similar to that observed for supercoil relaxation by other Type 1A topoisomerases) (121). However, the rate of positive supercoiling by *St*RG diminished abruptly at tensions of ∼0.3 pN. Since the torque required to form a plectoneme increases with the applied tension, it was proposed that the enzyme is unable to generate sufficient torque to induce supercoiling at tensions higher than 0.3 pN. By calculating the theoretical torque at a given force, it was estimated that the enzyme ceases to overwind DNA when opposed by a critical torque of ∼5 pN·nm. It was suggested that this may allow RGs to protect, but not overprotect, against thermal denaturation in (hyper)thermophiles (121).

In the second approach, Ogawa *et al.* probed the overwinding activity of *St*RG by allowing free rotation of fluorescently-labelled beads (through the use of a single vertically positioned magnet), such that overwinding could be detected directly by imaging the clockwise rotations of the bead (Figure 8C) (114,121). In this case, the torsional stress applied to the DNA during *St*RG activity depends only on the frictional torque associated with the bead, which is proportional to the rotational velocity of the bead. Using this assay, overwinding of DNA at 71°C by *St*RG was observed at tensions higher than 0.3 pN, namely 0.5 pN, owing to the fact that, at this tension, the frictional torque is much lower than 5 pN·nm. In addition, it was shown that *St*RG is inactive at temperatures ≤60°C, unless one or more 30-bp mismatches were introduced into the DNA (Figure 8D). Moreover, upon introduction of one 30-bp mismatch, the overwinding rate of *St*RG increased from 0.079 to 0.23 turns·s^–1^ (at 71°C and at 0.5 pN) (Figure 8D, cf. (i) and (ii)) (114,121). These findings indicate that binding and/or overwinding activity of *St*RG requires ssDNA, which is consistent with the catalytic mechanism of TopoI and TopoIII enzymes (as discussed earlier), as well as with the high affinity of the helicase domain of RGs for ssDNA (176). When increasing the number of 30-bp mismatches from one to two (which ensures two *St*RG binding sites), the overwinding rate of *St*RG increased 2-fold, as expected from the simultaneous activity of two enzymes (Figure 8D, cf. (ii) and (iii)) (114). However, no further increase in the overwinding rate was observed when using a substrate containing four 30-bp mismatches (Figure 8D, cf. (iii) and (iv)). This was attributed to the fact that the rotational velocity of the bead (induced by four enzymes acting on the DNA) results in a frictional torque that is sufficiently high to reduce the activity of each enzyme. By measuring the catalytic rate per enzyme as a function of frictional torque, a more direct estimate of the critical torque that restricts *St*RG activity was obtained, corresponding to ∼7 pN·nm (at 0.5 pN and 71°C) (114).

Further insights into the mechanism of RGs and the role of ATP were provided by Yang *et al.* using a magnetic tweezers assay similar to that shown in Figure 3A, i.e. where the rotation of the bead is controlled by a pair of horizontally-aligned magnets. The authors used this assay to monitor both the supercoil relaxation and DNA overwinding activity of *Sso*RG2 through the change in the DNA end-to-end length at a constant force (88). In contrast to *St*RG, *Sso*RG2 has been reported to be active on intact dsDNA at much lower temperatures than 71°C (86). In line with this, Yang *et al.* observed that, at a force of 0.2 pN and in the presence of ATP, *Sso*RG2 was able to relax negatively supercoiled DNA and subsequently overwind the DNA at temperatures as low as 45°C (88). Note that, here, both the tension and the temperature were sufficiently low such that the enzyme is assumed to interact with intact dsDNA. Moreover, when using a 1000-fold lower ATP concentration, the above magnetic tweezers assay enabled direct observation of the fundamental substeps of the catalytic cycle of *Sso*RG2 (Figure 8E and F). Under these conditions, *Sso*RG2 relaxed the negatively supercoiled DNA via steps of 1 *Lk* (consistent with the strand-passage mechanism of Type 1A topoisomerases), in a process that ultimately led to the formation of positive supercoiling (Figure 8E). Interestingly, the initial and final changes in the DNA end-to-end length were always twice the size as the change in length associated with strand passage. By studying the interaction of *Sso*RG2 with both negatively and positively supercoiled DNA in the absence of ATP (where the enzyme is catalytically inactive), it was revealed that the initial and final steps correspond to enzyme binding and unbinding, respectively. It was therefore deduced that enzyme binding induces a redistribution of writhe into twist by 2 *Lk*. Moreover, repeating the above experiment using a negatively supercoiled substrate containing a mismatch of either 5 or 10 bp yielded a redistribution of only ∼1.4 or ∼0.9 *Lk*, respectively. Taken together, these findings indicate that *Sso*RG2 binding results in the formation of a strand-separated bubble of ∼20 bp (88). In contrast, enzyme unbinding from positively supercoiled DNA resulted in a redistribution of twist into writhe by 2 *Lk* (and thus rewinding of the enzyme-mediated bubble).

In order to probe the influence of ATP binding and hydrolysis on the interactions of *Sso*RG2 with DNA, the above experiments were repeated using a mixture of low concentrations of AMP-PNP (to mimic the ATP bound state) and ATP. In this way, it was revealed that, upon *Sso*RG2 binding to the DNA, subsequent AMP-PNP binding (and thus ATP binding) results in partial rewinding of the strand-separated bubble by ∼10 bp (i.e. a redistribution of twist into writhe by 1 *Lk*) (Figure 8F). Furthermore, these experiments demonstrated that ATP hydrolysis (following ATP binding) re-established the 20-bp bubble. This step occurred simultaneously with a strand passage event, resulting in a linking number change of +1 *Lk* (Figure 8F) (88). Based on the smFRET measurements discussed earlier (122), the formation of a strand-separated bubble by *Sso*RG2 is likely correlated with the open state of the helicase domain, whereas the rewinding of the bubble is likely associated with the closed conformation of the helicase (which results from ATP binding (122)). The combined insights into the catalytic cycle of RGs obtained from the single-molecule studies described above are summarized in Figure 8G.

## CONCLUSION

As we have discussed in detail in this review, the application of single-molecule assays has yielded significant new insights into the molecular mechanisms and reaction kinetics of Type 1A topoisomerases, as well as the mechanistic interactions of these enzymes with partner proteins. Nonetheless, many questions remain unresolved. First, despite recent advances, the detailed molecular interactions that determine how the conformational changes in the DNA-enzyme complex influence the processivity and pause durations are still not fully understood. Moreover, most studies have so far been conducted on prokaryotic Type 1A topoisomerases, and much less is known about their eukaryotic homologues. It will be particularly important to gain greater mechanistic knowledge of the catalytic activity of human TopoIIIα and TopoIIIβ, not least as malfunction of these enzymes is implicated in several diseases (177–181). In the case of TopoIIIα, future studies will be required to reveal the mechanisms by which its binding partner RMI1 influences the catalytic activity. Furthermore, as TopoIIIβ has been reported to be required for efficient replication of positive-sense RNA viruses (182), future research on TopoIIIβ could aid the search for new potential anti-viral targets. In addition, it will be insightful to obtain a deeper mechanistic understanding of how TopoIII enzymes, RecQ-family helicases and ssDNA binding proteins act in concert to resolve late-replication and recombination intermediates.

It is anticipated that the single-molecule techniques outlined in this review, alongside other single-molecule assays, will play an important role in future studies of Type 1A topoisomerases. One application that could prove highly informative is the combination of single-molecule manipulation techniques with fluorescence microscopy (74,98,119,183). Visualizing the interaction of Type 1A topoisomerases with supercoiled DNA, for example using side-pulling magnetic tweezers in combination with TIRF microscopy (8,136), could provide greater understanding of the binding kinetics of these enzymes on different sequences and substrates. Fluorescence imaging experiments on supercoiled DNA can also be performed using a recently developed assay (termed Optical DNA Supercoiling) that enables negatively supercoiled DNA to be generated using dual-trap optical tweezers (145). This assay is advantageous as it can be readily combined with various fluorescence imaging modalities and (coupled with the use of a multi-channel flow cell) can facilitate efficient study of multi-protein interactions with negatively supercoiled DNA. Furthermore, quadruple-trap optical tweezers, which allow two DNA molecules to be independently manipulated using four optically-trapped beads (151,184–186), offer several key advantages for studying the decatenation activity of Type 1A topoisomerases. These include the ability to probe the influence of the crossover angle of braided DNA with much higher control compared to magnetic tweezers assays. Moreover, by exploiting the compatibility of optical trapping with multicolor fluorescence imaging and rapid buffer exchange, quadruple-trap optical tweezers could be employed to visualize the interaction of multi-protein complexes, such as the dissolvasome, on entangled DNA. Finally, it will be important to extend single-molecule approaches to probe more complex physiological substrates relevant for Type 1A topoisomerases, such as double Holliday junctions and (hemi)catenanes (74,187). Given the significant strides made over the past 20 years, coupled with the development of increasingly sophisticated single-molecule assays, there are exciting opportunities to obtain an even deeper understanding of the molecular mechanisms of Type 1A topoisomerases.

## DATA AVAILABILITY

All data presented are derived from previously published data sets, as indicated in the figure captions. Axes lines and tick marks have been superimposed directly over the original markings to ensure consistent line thickness. In some cases, we have additionally annotated the figures with arrows, text and lines to highlight specific relevant features in the data.
